# Editor's Letter-Box

**Published:** 1889-02-23

**Authors:** William J. Gant

**Affiliations:** Secretary. The Hospital, Hastings


### EDITOR'S LETTER-BOX.

[Our Correspondents are reminded that prolixity is a great bar to publication, and that brevity of style and conciseness of statement greatly facilitate early insertion.]

SIR,—I observe in your number of February 9th, a paragraph headed "A Lack of Courtesy," being a complaint made by a member of The Hospital Association, that a nurse of some years' standing was refused by the porter to be allowed to go over this hospital, although she had presented herself at a quarter to three o'clock on a Friday afternoon, and consequently on the right day and within the hour for showing visitors over. I have inquired into the matter, and am assured by the porter that he does not recollect the particular case, but that he is certain that he has never taken upon himself to refuse admittance to a visitor on any occasion, and that when a person has called at an irregular hour he has communicated with the matron or house surgeon; this the matron and house surgeon confirm. The porter thinks the nurse's watch must have been at fault, as between three and four patients' friends are visiting the hospital, and that hour would certainly not be convenient; whilst at a quarter before three o'clock it would be otherwise. I take this opportunity of stating that nurses, no less than others doing hospital work, are welcome to go over the hospital, and that I am sure the matron will be glad to give them any information in her power. WILLIAM J. GANT, Secretary.

The Hospital, Hastings, February 16th, 1889.

[We can confirm the secretary's statement so far as our own experience is concerned. On visiting the Hastings Hospital all possible attention was shown us both by the matron and other officials.—ED. *T.H.*]

